# TNF-α and IGF1 modify the microRNA signature in skeletal muscle cell differentiation

**DOI:** 10.1186/s12964-015-0083-0

**Published:** 2015-01-29

**Authors:** Swanhild U Meyer, Christian Thirion, Anna Polesskaya, Stefan Bauersachs, Sebastian Kaiser, Sabine Krause, Michael W Pfaffl

**Affiliations:** Physiology Weihenstephan, ZIEL Research Center for Nutrition and Food Sciences, Technische Universität München, Weihenstephaner Berg 3, D-85354 Freising, Germany; SIRION Biotech GmbH, Am Klopferspitz 19, 82152 Martinsried, Germany; CNRS FRE 3377, Univ. Paris-Sud, CEA Saclay, iBiTec-S/ SBIGeM, F-91191 Gif-sur-Yvette, France; Laboratory for Functional Genome Analysis (LAFUGA), Gene Center, LMU Munich, Feodor-Lynen-Str. 25, 81377 Munich, Germany; Department of Statistics, Ludwig-Maximilians-Universität München, Ludwigstr. 33, 80539 Munich, Germany; Friedrich-Baur-Institute, Department of Neurology, Ludwig-Maximilians-Universität München, Marchioninistr. 17, 81377 Munich, Germany; Current address: ETH Zurich, Institute of Agricultural Sciences, Animal Physiology, Universitätstrasse 2 / LFW B 58.1, 8092 Zurich, Switzerland

**Keywords:** microRNA, TNF-α, IGF1, Skeletal muscle cell, Expression profiling, Myoblast differentiation, Human, Murine, miRNA biogenesis, MAPK

## Abstract

**Background:**

Elevated levels of the inflammatory cytokine TNF-α are common in chronic diseases or inherited or degenerative muscle disorders and can lead to muscle wasting. By contrast, IGF1 has a growth promoting effect on skeletal muscle. The molecular mechanisms mediating the effect of TNF-α and IGF1 on muscle cell differentiation are not completely understood. Muscle cell proliferation and differentiation are regulated by microRNAs (miRNAs) which play a dominant role in this process. This study aims at elucidating how TNF-α or IGF1 regulate microRNA expression to affect myoblast differentiation and myotube formation.

**Results:**

In this study, we analyzed the impact of TNF-α or IGF1 treatment on miRNA expression in myogenic cells. Results reveal that i) TNF-α and IGF1 regulate miRNA expression during skeletal muscle cell differentiation *in vitro*, ii) microRNA targets can mediate the negative effect of TNF-α on fusion capacity of skeletal myoblasts by targeting genes associated with axon guidance, MAPK signalling, focal adhesion, and neurotrophin signalling pathway, iii) inhibition of miR-155 in combination with overexpression of miR-503 partially abrogates the inhibitory effect of TNF-α on myotube formation, and iv) MAPK/ERK inhibition might participate in modulating the effect of TNF-α and IGF1 on miRNA abundance.

**Conclusions:**

The inhibitory effects of TNF-α or the growth promoting effects of IGF1 on skeletal muscle differentiation include the deregulation of known muscle-regulatory miRNAs as well as miRNAs which have not yet been associated with skeletal muscle differentiation or response to TNF-α or IGF1. This study indicates that miRNAs are mediators of the inhibitory effect of TNF-α on myoblast differentiation. We show that intervention at the miRNA level can ameliorate the negative effect of TNF-α by promoting myoblast differentiation. Moreover, we cautiously suggest that TNF-α or IGF1 modulate the miRNA biogenesis of some miRNAs via MAPK/ERK signalling. Finally, this study identifies indicative biomarkers of myoblast differentiation and cytokine influence and points to novel RNA targets.

**Electronic supplementary material:**

The online version of this article (doi:10.1186/s12964-015-0083-0) contains supplementary material, which is available to authorized users.

## Background

Detailed understanding of the molecular mechanisms through which external stimuli such as inflammatory cytokines or growth factors modulate skeletal muscle differentiation is vital for the appreciation of muscle regeneration. After skeletal muscle injury, differentiation of myoblasts into myotubes and maturation into myofibers is essential for muscle repair which involves multiple steps such as myoblast proliferation, migration, alignment, recognition, adhesion, cell fusion, and reorganization of the extra cellular matrix [1]. IGF1 can promote muscle differentiation [2] and can enhance muscle maintenance and repair [3]. The underlying regulatory mechanism is not completely understood. However, a better understanding of IGF1 signalling in skeletal muscle is important for therapeutic application [3]. In many chronic diseases or muscular disorders, inflammatory cytokine levels such as TNF-α are elevated [4-6]. TNF-α is associated with cachectic muscle wasting [7] and inhibits skeletal muscle differentiation at higher concentrations [8].

Post-transcriptional regulators of gene expression, such as microRNAs (miRNAs) are increasingly recognized as differentially regulating protein expression and playing a key role in skeletal muscle differentiation, repair and maintenance [9,10]. Moreover, miRNA expression is deregulated in many muscular disorders [11] in which secondary processes involve persistent inflammation and impaired muscle regeneration [12]. Various therapeutic strategies are currently being investigated to promote skeletal muscle growth and regeneration [13]. Nuclear factor kappa B (NF-κB) links the inhibition of muscle differentiation by inflammatory cytokines [14] such as TNF-α to the negative regulation of myogenic regulatory factors (MRFs) [15,16], while IGF1 increases MRF expression [17]. As some muscle-regulatory miRNAs are regulated by these MRFs [18], we hypothesized that miRNAs may be potent mediators of TNF-α and IGF1 signalling during myoblast differentiation. Consistent with this hypothesis, miRNAs expression is affected during skeletal muscle wasting induced by TWEAK [19], a member of the TNF superfamily with multiple biological activities [20]. However, the transcriptomic response and the regulation of miRNAs in skeletal myoblast differentiation upon TNF-α or IGF1 exposure have, to the best of our knowledge, not yet been described. Therefore, we assessed the effects of TNF-α and IGF1 on differentiation of skeletal myoblasts *in vitro* and analyzed the miRNA expression status in early skeletal muscle cell differentiation revealing that miRNA expression is modulated by these cytokines, possibly by MAPK/ERK signalling. Furthermore, functional miRNA studies indicated that the impact of TNF-α and IGF1 on myotube formation involved with specific miRNA activity. Finally, our study provides valuable insights into a more detailed understanding of how TNF-α and IGF1 effects are mediated via miRNA expression.

## Results

### Myoblast differentiation regulates miRNAs which have not been associated with skeletal muscle physiology

**Figure 1:**
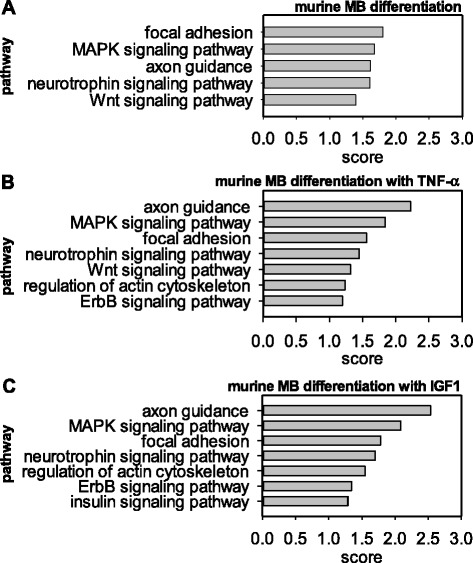
**Predicted targets of murine miRNAs associated with myogenic differentiation, TNF-α or IGF1 response are enriched in specific pathways.** Selected KEGG pathways within the top 10 enriched KEGG pathways of predicted miRNA targets in mouse for **(A)** targets of miRNAs which are differentially regulated during differentiation, **(B)** during differentiation with TNF-α exposure, or **(C)** IGF1 treatment.

Differentiation of murine cell line and human primary skeletal myoblasts was induced *in vitro* by serum withdrawal. After 24 hours we studied the impact of myogenic differentiation on miRNA expression. We identified a significant regulation of miRNAs which are among the 15% most abundantly expressed species in myoblasts or myotubes (Table 1, complete list in Additional file 1A,B). MiRNAs which are at least four-fold upregulated or downregulated during human or murine skeletal myoblast differentiation are listed in Table 1. We confirmed upregulation of miR-1, miR-133a, miR-133b and miR-206, which have previously been extensively implicated in skeletal muscle development and function [21]. A subset of seven differentially regulated miRNAs was simultaneously retrieved in the human and the murine skeletal muscle cells: miR-1, miR-133a-3p, miR-133b, miR-135a-3p, miR-206, miR-450b-5p, miR-451a, and miR-497-5p (Table 1A,B, Additional file 1A,B). Moreover, we identified several miRNAs which, to the best of our knowledge, have not been detected in skeletal myoblast differentiation before, such as mmu-miR-202-3p, mmu-miR-344-3p, mmu-miR-376b-5p, mmu-miR-409-3p, and hsa-miR-216a-5p (Additional file 1A,B). In addition, our data revealed regulation of miRNAs for which only an isoform or the corresponding 3p or 5p miRNA have previously been described in myoblast differentiation such as mmu-miR-322-3p (Additional file 1A,B). *In vitro* myoblast differentiation also involved miRNAs which are differentially expressed in primary muscular disorders (e.g. mmu-miR-146a-5p, mmu-miR-335-5p, hsa-miR-299-5p [11]), aged muscle (e.g. mmu-miR-434-5p [22], mmu-miR-451a [23]), or insulin resistant muscle in diabetes patients (e.g. has-miR-15a-5p [24], mmu-miR-503-5p [25]) (Additional file 1A, B). Target prediction and enrichment analysis for differentiation-related miRNAs revealed predicted targets enriched for functional annotations including the spliceosome, RNA degradation, peroxisome proliferator-activated receptor (PPAR) signalling pathway, and neuroactive ligand-receptor interaction (Additional file 2A, D). Moreover, predicted miRNA targets were overrepresented in KEGG pathways such as focal adhesion, MAPK signalling, axon guidance, neurotrophin signalling, and Wnt signalling (Figure 1A, Additional file 3A; Additional file 4A,B).

**Table 1:** **miRNA signatures of myoblast differentiation and TNF-α or IGF1 treatment**

**A**							
		**TLDA**			**Agilent**		
**Human miRNA (hsa-miR)**		**Differentiation ΔΔCt**		**TNFα ΔΔCt**	**Differentiation log2**		**TNFα log2**
**1**		**2.0**		−0.2	**1.1**		**−1.1**
**133b**	●●	**2.3**		**−3.2**	**1.9**		**−2.0**
198		1.1		**−2.1**	n.d.		n.d.
**206**	●●	**2.2**		**−2.1**	**2.0**		**−2.1**
216a-5p	●●	**−4.4**		**5.5**	n.d.		n.d.
**218-5p**		0.8		**2.4**	0.2		**2.4**
299-5p		**2.2**		−0.8	0.3		−0.2
433	●	**2.7**		**−2.1**	n.d.		n.d.
451a		-		-	**−2.6**		0.1
**B**							
		**qPCR array**			**microarray**		
**murine miRNA (mmu-miR)**		**Differentiation ΔΔCt**	**TNFα ΔΔCt**	**IGF1 ΔΔCt**	**Differentiation log2**	**TNFα log2**	**IGF1 log2**
125b-2-3p		**2.0**	−0.9	−0.7	n.d.	n.d.	n.d.
129-5p		**−2.4**	−0.6	0.1	n.d.	n.d.	n.d.
**133a-3p**	●●	**1.1**	−0.3	−0.1	**2.1**	−0.5	0.3
133a-5p		-	-	-	**3.4**	**−0.8**	0.5
**133b-3p**	●●	**2.7**	**−0.6**	0.2	**2.4**	−0.5	0.4
137-3p		1.3	**−2.0**	−0.6	n.d.	n.d.	n.d.
146a-5p		0.0	**3.6**	**−1.2**	n.d.	n.d.	n.d.
**202-3p**		**−4.1**	**5.6**	4.4	−1.2	**1.2**	−0.1
**206-3p**	●●	**2.6**	−0.3	0.1	**2.5**	−0.3	0.5
**322-3p**	●	**2.5**	**−1.3**	−0.1	**1.8**	**−1.0**	0.2
330-3p		−0.7	−2.2	**−6.0**	**2.7**	**−1.0**	0.0
**335-3p**	●●	**2.5**	**−0.9**	0.0	2.7	−1.0	0.0
**351-5p**		**2.1**	**−0.8**	**0.7**	**2.3**	**−0.9**	0.4
434-5p		0.8	**−2.1**	0.3	n.d.	n.d.	n.d.
451a		**−5.2**	−4.3	1.2	n.d.	n.d.	n.d.
468-3p		-	-	-	−0.8	**−1.4**	**−2.0**
**483-3p**	●	**2.6**	0.4	0.2	**0.6**	0.4	0.3
503-3p		**2.4**	−0.8	0.3	n.d.	n.d.	n.d.
**503-5p**		**1.4**	**−1.3**	0.0	**2.3**	**−1.7**	0.1
542-3p		**1.3**	**−1.5**	0.2	2.6	−2.4	−0.7

Differential miRNA expression is depicted as deltadelta Cq or log2 (signal intensity) for (A) human and (B) murine miRNA expression profiling by qPCR or microarrays for the effect of differentiation and TNF-α or IGF1 treatment. miRNAs with p-values < 0.05 and expression regulation > 4-fold in at least one test series are depicted. Bold numbers indicate significant (p < 0.05) and > 2-fold regulation. Bold miRNAs imply cross-platform validation: The respective miRNAs were significantly regulated in at least one of the treatment effects on both platforms (TLDA and Agilent). One black circle denotes if the miRNA is among the 25% most expressed miRNAs in at least one of the treatment groups on the qPCR array. Two black circles depict miRNAs which are among the 15% most expressed miRNAs in at least one of the treatment groups on the qPCR array. Some miRNAs could not be detected (n.d.) or were not present (dash) on one of the platforms.

### TNF-α treatment specifically regulates and counteracts miRNA expression during skeletal myoblast differentiation

In a next set of experiments, we examined the differential miRNA expression in TNF-α treated, differentiated primary human and murine cell line myoblasts *in vitro*. TNF-α exposure differentially regulated miRNAs in human and mouse skeletal muscle cells which are among the 15% most abundantly expressed miRNAs (Table 1, Additional file 1A,B). We identified miRNAs which are significantly upregulated (e.g. mmu-miR-146a-5p, hsa-miR-15a-5p, mmu-miR-202-3p, hsa-miR-216a-5p, hsa-miR-218-5p, mmu-miR-721) or downregulated by TNF-α during myogenic differentiation (e.g. mmu-miR-133b-3p, mmu-miR-137-3p, hsa-miR-149-5p, hsa-miR-198, hsa-miR-206, mmu-miR-409-3p, hsa-miR-433, mmu-miR-434-5p, mmu-miR-503-5p, and mmu-miR-542-3p) (Table 1, Additional file 1A,B). A subset of miRNA was inversely regulated more than two-fold each during myogenic differentiation and TNF-α treatment: hsa-miR-1, mmu-miR-133a-3p, hsa-miR-133b, mmu-miR-133b-3p, mmu-miR-202-3p, hsa-miR-206, mmu-miR-206-3p, hsa-miR-216, mmu-miR-322-3p, mmu-miR-322-5p, mmu-miR-33-3p, mmu-miR-335-3p, mmu-miR-335-5p, mmu-miR-409-3p, hsa-miR-433, mmu-miR-450a-5p, mmu-miR-503-5p, and mmu-miR-877 (Table 1, Additional file 1A,B). The regulation of miRNAs by TNF-α coincided with an impaired fusion capacity demonstrated by a significantly reduced fusion index (Additional file 5). Moreover, functional annotations of predicted miRNA targets in the differentiation and the TNF-α treatment group include RNA degradation and neuroactive ligand-receptor interaction (Additional files 2B,E; 4A,B). Predicted targets of TNF-α-regulated miRNAs are enriched in KEGG pathways such as axon guidance, MAPK signalling, focal adhesion, neurotrophin, Wnt and ErbB signalling pathways (Figure 1B, Additional files 3B; 4A,B). Common pathway enrichments and functional annotations of predicted targets of differentiation and TNF-α-regulated miRNAs triggers the speculation that the role of TNF-α in counteracting differentiation-associated miRNAs might predominate over TNF-α specific induced or suppressed miRNA regulation.

### IGF1 treatment both promotes and inversely regulates myoblast differentiation-like miRNA expression patterns

IGF1 treatment of murine differentiating skeletal muscle cells resulted in upregulation (e.g. mmu-miR-7b-5p, mmu-miR-363-3p, mmu-miR-680) and downregulation (e.g. mmu-miR-146a-5p, mmu-miR-148a-3p, mmu-miR-330-3p, mmu-miR-468-3p) of miRNA subsets (Table 1, Additional file 1A, B). IGF1 enhanced miRNA expression seen in myogenic differentiation in the case of mmu-miR-351-3p, mmu-miR-139-5p, mmu-miR-450b-5p (Table 1, Additional file 1A, B). By contrast, mmu-miR-330-3p expression was substantially decreased by IGF1 exposition. Moreover, IGF1 treatment inversely regulated the expression of mmu-miR-7b-5p, mmu-miR-33-3p, and mmu-miR-98-5p as compared to the expression pattern seen in differentiating control myoblasts (Table 1, Additional file 1A,B). Regulation of miRNA expression by IGF1 coincided with a positive effect on fusion capacity, as the fusion index was increased by more than 30% (Additional file 5). Furthermore, microRNAs differentially expressed in response to IGF1 treatment showed predicted targets which are enriched for functional annotations such as oxidative phosphorylation, neurodegenerative diseases, axon guidance, and muscle contraction (Additional files 2C, 4B). Besides, target prediction analysis of IGF1-regulated miRNAs revealed an enrichment of targets in KEGG pathways similar to control myoblast differentiation and TNF-α treatment including axon guidance, MAPK signalling, focal adhesion, and neurotrophin signalling (Figure 1C). Moreover, IGF1 regulated miRNAs are predicted to target insulin signalling pathway-associated genes (Figure 1C).

### MicroRNA signatures are indicative biomarkers for myoblast differentiation and TNF-α or IGF1 exposure

**Figure 2:**
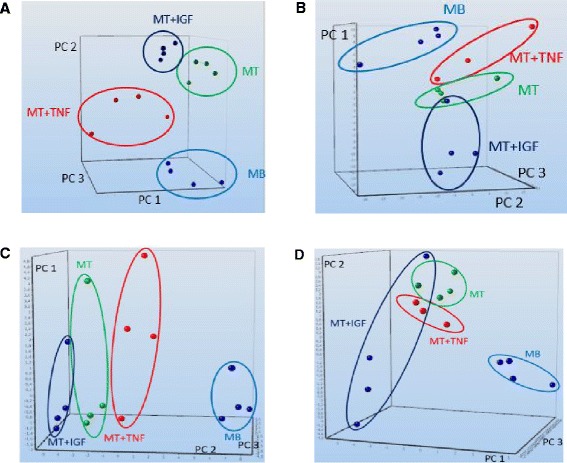
**Principal component analyses of miRNA in early skeletal myoblast differentiation and TNF-α or IGF1 treatment.** Principal component analyses of murine miRNA expression profiling data after 24 h of induction differentiation and TNF-α or IGF1 treatment. Principal component analysis reveals separation of treatment groups for **(A)** microarray and **(B)** qPCR data. Dynamic principal component analysis (group selection myoblasts) identifies the most relevant subset of miRNAs which can describe the treatment effects and separate the effects by principal components for **(C)** microarray and **(D)** qPCR data. Axes depict principal component 1 (PC 1), principal component 2 (PC 2), and principal component 3 (PC 3).

The regulation of miRNA expression by TNF-α and IGF1 treatment of differentiating murine skeletal muscle cells appears in separation of treatment groups by hierarchical cluster analysis (Additional file 6A and B) or principal component analysis (Figure 2A,B). The distance between treatment groups depended on the profiling platform used as well as the clustering approach. We applied dynamic principal component analysis to identify the most relevant miRNAs explaining our observations (Figure 2C,D). However, separation of treatment groups became less clear or even disappeared between control myotubes and TNF-α or IGF1-treated myotubes, whereas myoblasts always separated from the differentiation effects. Five miRNAs which are in the subset of miRNAs derived from dynamic principal component analysis were identified by the miRNA microarray as well as the qPCR-platform: mmu-miR-133b-3p, mmu-miR-188-3p, mmu-miR-206-3p, mmu-miR-335-3p, mmu-miR-351-3p (Table 2). In conclusion, variable selection by dynamic principal component analysis revealed distinct miRNAs identified on different platforms which might be indicative biomarkers for myoblast differentiation.

**Table 2:** **miRNA subsets derived from dynamic principal component analysis**

**miRNA 24 h (microarray)**	**miRNA 24 h (qPCR)**
miR-1a-3p	miR-124
miR-128-3p	miR-129-5p
miR-1306-3p	**miR-133b-3p**
miR-133a-3p	miR-139-3p
miR-133a-5p	miR-141-3p
**miR-133b-3p**	miR-142-5p
miR-135a-1-3p	**miR-188-3p**
miR-139-5p	miR-197
**miR-188-3p**	miR-19a-5p
miR-1894-3p	miR-200a-3p
miR-1971	**miR-206-3p**
**miR-206-3p**	miR-211
miR-20a-3p	miR-292-3p
miR-21a-3p	miR-302b
miR-29b	miR-329
miR-3107-5p	**miR-335-3p**
miR-32-5p	**miR-351-5p**
miR-322-5p	miR-367-3p
miR-322-3p	miR-369-3p
**miR-335-3p**	miR-451a
miR-335-5p	miR-452-5p
**miR-351-5p**	miR-483-3p
miR-382-5p	miR-878-5p
miR-450b-3p	miR-881-3p
miR-450b-5p	
miR-467f	
miR-468-3p	
miR-483-3p	
miR-500-3p	
miR-501-3p	
miR-503-5p	
miR-532-3p	
miR-542-3p	
miR-542-5p	
miR-598-3p	
miR-696	
miR-99b-3p	

Murine miRNAs which are sufficient for identifying principal components as shown in Figure 2B. Bold miRNAs are present in the microarray as well as the qPCR subset.

### MiRNAs potentially mediate the inhibitory effect of TNF-α on myoblast differentiation

**Figure 3:**
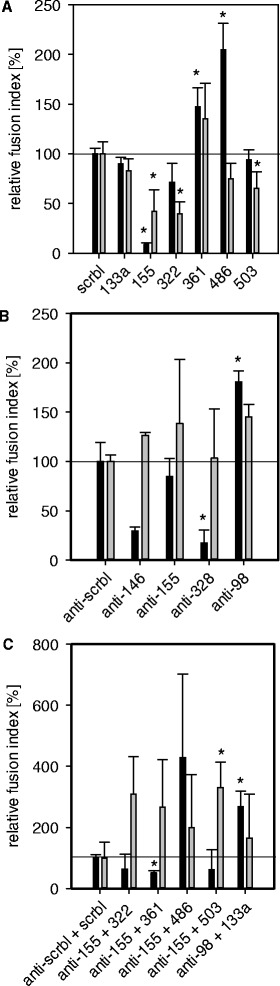
**Functional analysis of miRNAs in human myoblast differentiation and TNF-α treatment.** Relative fusion indices for miRNA mimics or miRNA inhibitor transfections into human LHCN myoblasts in the differentiation medium (black bars) or differentiation medium with TNF-α supplementation (grey bars). **(A)** Transfection of 25 nM miRNA mimics or scrambled control miRNA (scrbl), **(B)** 50 nM miRNA inhibitors or scrambled control inhibitors (anti-scrbl), and **(C)** a combination of 75 nM miRNA inhibitor and 25 nM miRNA mimic or respective controls. Samples with specific miRNAs or inhibitors are shown relative to the respective scrambled reference which was set to 100% fusion index. Significant differences (p > 0.05) of relative fusion indices are marked by an asterisk.

**Figure 4:**
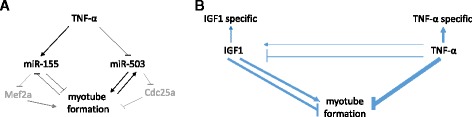
**Intervention at the miRNA level can rescue the effect of TNF-α on myotube formation. (A)** TNF-α partly mediates its negative effect on myotube formation through interfering at the miRNA level. TNF-α stimulates expression of miR-155 which is downregulated during myoblast differentiation. Overexpression of miR-155 blocked myotube formation. TNF-α downregulated miR-503 expression during myoblast differentiation. miR-503 expression was upregulated during myotube formation. Simultaneous inhibition of miR-155 and overexpression of miR-503 rescued the inhibitory effect of TNF-α on myotube formation. Grey indicates validated targets Mef2a [36] and Cdc25a [38]. **(B)** Schematic overview of the regulation of miRNA expression by TNF-α or IGF1 during skeletal myoblast differentiation. The width of the blue arrows or lines indicate the relative quantity of miRNAs regulated by TNF-α or IGF1 in skeletal myoblast differentiation. TNF-α mainly inversely regulated miRNAs expression associated with myoblast differentiation. IGF1 promoted and antagonised myogenesis associated miRNA expression. Few miRNAs are specifically regulated by TNF-α or IGF1, and in turn, part of these miRNAs are counteracted by TNF-α or IGF1.

Functional miRNA analyses were performed in a human skeletal muscle precursor cell line, LHCN [26] which showed strong sensitivity to the repressive effect of TNF-α upon myotube formation *in vitro* (Additional file 5). We aimed at rescuing the inhibitory effect of TNF-α on myoblast fusion efficiency by i) overexpression of miRNAs which were upregulated during differentiation in murine PMI28 cells, primary human skeletal muscle cells (Table 1B, Additional file 1A,B) or in human LHCN muscle cells [10] or ii) by inhibition of miRNAs which are downregulated during differentiation but upregulated due to TNF-α or iii) by promoting differentiation-associated miRNA patterns by a combination of overexpression and inhibition of miRNAs which are inversely regulated during control differentiation and TNF-α treatment. However, overexpression or inhibition of selected miRNAs did not outweigh the negative effect of TNF-α on fusion capacity (Figure 3A,B). However, combined inhibition of miR-155 and overexpression of miR-503 ameliorated the negative effect of TNF-α on differentiation (Figures 3C, 4A). Overexpression of hsa-miR-361, hsa-miR-486 or inhibition of hsa-miR-98 alone or in combination with hsa-miR-133a overexpression enhanced fusion capacity in control myoblasts significantly but was not powerful enough to rescue the TNF-α effect (Figure 3A,B,C). Some miRNAs or inhibitors resulted in partial detachment of the cell layer, leading to high standard deviations.

### MAPK/ERK may participate in modulating the effect of TNF-α and IGF1 on miRNA abundance

According to a study by Paroo et al. [27] TAR RNA binding protein (TRBP) phosphorylation by MAPK/ERK elevated cell growth, possibly as a consequence of upregulating the biogenesis of growth-stimulatory miRNAs and decreasing miRNAs which suppress proliferation. Both, TNF-α and IGF1 activate MAPK/ERK signalling [28-30], and resemble two stimuli with inverse outcome on skeletal myoblast differentiation. Thus, we hypothesized that MAPK/ERK activation by opposing extracellular signals affect miRNA biogenesis in a different manner (Additional file 7). Therefore, we assessed the impact of 24 h treatments with TNF-α, IGF1, or pharmacological MAPK/ERK inhibition, as well as simultaneous combinations of TNF-α and MAPK/ERK inhibition or IGF1 and MAPK/ERK inhibition on the expression of myogenic differentiation markers in murine skeletal muscle cells. Our study evaluated the effect on the mRNA expression of myogenin (Myog), myocyte-specific enhancing factor 2C (Mef2c), myogenic factor 5 (Myf5), and myosin heavy chain 1 (Myh1) and the effect on myogenic differentiation-regulated miRNAs such as miR-1, miR-133a, and miR-206 (Additional file 8A-D). Expression analyses of mature and precursor miRNAs in murine skeletal myoblasts cautiously support the hypothesis that biogenesis of mature mmu-miR-1 and mmu-miR-133a can be regulated by TNF-α, IGF1, and MAPK/ERK (Additional file 8A, B). The effect on miRNA processing seems to be i) miRNA-specific, as precursor and mature forms of, for example, mmu-miR-206 were similarly regulated (Additional file 8C) rather indicating transcriptional regulation and ii) precursor specific, as mmu-miR-1-1 and mmu-miR-1-2 or mmu-miR-133a-1 and mmu-miR-133a-2 are distinctly affected (Additional file 8A, B). MAPK/ERK might participate in mediating the effect of TNF-α and IGF1 on mature miRNA abundance, as the effect of TNF-α and IGF1 on miRNA expression was modulated in cells treated with MAPK/ERK inhibitor (Additional file 8A-C). Firstly, mmu-miR-1 or mmu-miR-206 downregulation by TNF-α was diminished when MAPK/ERK inhibitor was applied and myogenic marker expression was unaffected. Secondly, IGF1 treatment in combination with MAPK/ERK- inhibitor treatment might have a synergistic positive effect on mmu-miR-1, mmu-miR-133a, and mmu-miR-206 expression, as well as on myogenic marker expression myogenin, Mef2c, Myf5, and Myh1.

To formally confirm treatment efficiency of U0126, a widely used inhibitor of the MAPK/ERK pathway, in our 24 h-inhibition experiment, we relied on our observation of the predicted indirect effect on synergistic upregulation of myogenin, Mef2c, Myf5, and Myh1 mRNA expression along with IGF treatment and the decreased downregulation of Myf5 and Myh1 in combination with TNF-α exposure (Additional file 8D). It is well established that the regulation of e.g. myogenin mRNA is highly dynamic, in particular within the first 24 h of robust myogenic induction by ITS (insulin, transferrin, selenium) treatment [31]. The regulation of myogenin mRNA stability is provided by a scheduled expression of RNA binding proteins specifically determining its half-life within a range of 2.5–10.5 h throughout the very early myogenic differentiation period for 24 h. Half-life of myogenin mRNA appeared to be maximal after about 20 h [31].

By contrast, the predicted half-life of myogenin protein is 20 h. Rapid changes of the mRNA level within the first 24 h might not be reflected at the protein level. For these technical reasons, we were reluctant to perform western blotting to assess the protein expression level of myogenin in our 24 h inhibition experiment. A series of time points measured at hourly intervals will be required to follow substantial short-term alterations both at the mRNA and protein level.

## Discussion

This study aimed at elucidating how cytokines involved in muscle cell proliferation and differentiation affect myotube formation by modulating miRNA expression *in vitro*. Consistent with previously published data, we detected miR-1, miR-133 and miR-206 regulation and corroborated that primary human and murine skeletal muscle cells represent *bona fide* cell culture models for myogenic differentiation. Our miRNA profiling data reveals that TNF-α and IGF1, two extracellular stimuli with opposed effects on myoblast differentiation capacity, induced, antagonised or promoted the miRNA expression pattern of human and murine skeletal muscle cell differentiation. TNF-α mainly inversely regulates miRNAs expression associated with myoblast differentiation while IGF1 both, promotes and antagonises myogenesis associated miRNA expression (Figure 4B). Target predictions indicate that miRNA functions might be enriched in the regulation of axon guidance, focal adhesion, neurotrophin, and MAPK signalling.

We also identified several miRNAs which have been associated with primary muscular disorders, aged muscle, sarcopenia, response to inflammatory stimuli, dexamethasone treatment or muscular insulin resistance. This is in line with the suggested negative effect of TNF-α on muscle regeneration in Duchenne muscular dystrophy [32] or in sarcopenia [33].

In addition, our data revealed significant differential regulation of novel miRNAs which, to the best of our knowledge, have not yet been associated with skeletal muscle cell differentiation or the effect of TNF-α on skeletal muscle cells before (Table 1, Additional file 1A,B). Moreover, TNF-α inversely regulated the expression of skeletal muscle differentiation-promoting miRNAs (e.g. mmu-miRNA-133b-3p, hsa-miR-133b) which suggests that the inhibitory effect of TNF-α on myoblast differentiation might be mediated in part by interfering with myogenic differentiation-associated miRNA expression. We identified TNF-α-deregulated miRNAs which have known pro or anti-myogenic functions, such as the miRNA family members miR-1 and miR-133a/-133b [34,35], miR-155 [9,36], miR-206 [34,37], miR-322-5p [38], miR-433 [39] and miR-503-5p [38]. On the other hand, we also identified several novel skeletal myoblast differentiation-associated miRNAs counteracted by TNF-α, which have not been described in this context, such as miR-216 [40], miR-409-3p [41], miR-877, or miR-202-3p. It has been suggested that miR-202-3p is an inhibitor of proliferation [42] and may function as a tumor suppressor [43] by targeting Gli1 [44] which mediates Myf5 expression during somitogenesis [45]. Early myogenic development during embryogenesis is in good agreement with a role of miR-202-3p in terminal myoblast differentiation. Moreover, miR-202-3p is a potential factor for the repression of PPAR-gamma coactivator 1 in acute inflammation [46]. It remains to be determined whether TNF-α induced upregulation of miR-202-3p in myoblast differentiation contributes to an impaired differentiation phenotype. Furthermore, we identified TNF-α and IGF1 as two stimuli with inverse effects on miR-450b-5p expression during muscle differentiation. So far, miR-450b-5p downregulation has been linked to the diabetic heart [47] but not to skeletal muscle cell differentiation.

Based on dynamic principal component analyses, we postulate that a subset of myogenic differentiation-associated miRNAs (mmu-miR-133b-3p, mmu-miR-188-3p, mmu-miR-206-3p, mmu-miR-335-3p, and mmu-miR-351-5p), of which most (four out of five) are deregulated by TNF-α exposure, are indicators for the negative effect of TNF-α myoblast differentiation. We speculate that those miRNAs could be valuable biomarkers of the regenerative status of the muscle in inflammatory contexts, or during the therapeutic response to anti-inflammatory substances such as dexamethasone, which has been shown to regulate miR-351 [48].

It is of particular interest to identify miRNAs which are inversely regulated in control myotube formation or IGF1 treatment compared to TNF-α treated differentiating myoblasts, as these miRNAs might be potential mediators of the negative effect of TNF-α on myoblast fusion. Supporting this hypothesis, we find that predicted targets of TNF-α regulated miRNAs are retrieved in pathways important for the control of myoblast differentiation. This indicates that inversely regulating the expression of differentiation-linked miRNAs might predominate the impact of TNF-α on the miRNA level over miRNAs which are responsive to TNF-α only. Indeed, shifting the expression of hsa-miR-155 and hsa-miR-503, two miRNAs inversely regulated by TNF-α, towards the level seen in myoblast differentiation, rescued the inhibitory effect of TNF-α on myotube formation (Figure 4A). This finding might be interesting from a therapeutic point of view, as miR-155 is upregulated in inflammatory condition such as rheumatoid arthritis [49] or various primary muscle disorders [11]. Dexamethasone treatment upregulated miR-503 expression in myotubes, but induced atrophy [48]. Our phenotypic fusion index assays suggested that further therapeutic exploitation of simultaneous miR-155 inhibition in combination with miR-503 mimics bears potential for treatment of immune-mediated muscle atrophy.

A subset of miRNAs which was regulated in both, the human primary skeletal muscle cells and the murine skeletal muscle cell line, could indicate robust association with myoblast differentiation across different model systems. These miRNA families have been associated with TWEAK exposed myotubes, aged muscle or Duchenne muscular dystrophy (DMD) (see Additional file 1A,B). miR-450b-5p has not been associated with the differentiation or pathogenesis of skeletal muscle cells, however, our data suggests biological significance in myoblast differentiation and response to TNF-α exposure. Murine myoblasts showed a higher number of differentially expressed miRNAs, probably due to species differences, due to a higher differentiation velocity of murine cells compared to human primary myoblasts, or to a higher biological variance of human primary cells compared to the murine cell line. Moreover, different detection patterns might be the result of technical differences of profiling technology and the respective underlying miRBase version. Several miRNAs were significantly regulated on the qPCR platform, but not detected on the microarray. This discrepancy may reflect higher sensitivity with real-time qPCR or suboptimal probe design in microarray chips for the assessed cell culture systems.

Finally, we speculate that MAPK/ERK might participate in mediating the effect of TNF-α and IGF1 on miRNA abundance. It has been shown that IGF1 initiates the Raf/MEK/ERK axis [50] and that TNF-α leads to MAP kinase activation, although with a very different profile of activation as compared to IGF1 [51]. Furthermore, it has been reported that MAPK/ERK activity modulates miRNA biogenesis in cancer cell lines by phosphorylating TAR RNA binding protein (TRBP) and stabilizing the Dicer-TRBP complex which enhances mature miRNA production, whereas pharmacological inhibition of MAPK/ERK decreases growth-promoting miRNAs and increased growth-suppressive miRNAs [27]. Our data cautiously indicates that maturation of distinct muscle-enriched miRNAs might be regulated by MAPK/ERK, TNF-α and IGF1, and that MAPK/ERK inhibition rescues the inhibitory effect of TNF-α and enhances the stimulating effect of IGF1 on some miRNAs. Therefore, MAPK/ERK inhibition in skeletal muscle differentiation, acting at the level of TNF-α and IGF1 regulated miRNAs, might have therapeutic significance.

In addition, our findings suggest that ERK inhibition can counteract the decrease of myogenic differentiation markers, as has been shown by Penna et al. [52]. However, our study describes for the first time the potential role of MAPK/ERK in modulating the effect of TNF-α and IGF1 at the miRNA expression or miRNA biogenesis level in skeletal myoblasts *in vitro*.

In summary, our findings confirm the hypothesis that TNF-α exposure impairs skeletal myoblast differentiation by interference at the miRNA level. Furthermore, IGF1 treatment regulates miRNA expression during myogenesis *in vitro*.

## Conclusions

The study of miRNA expression in skeletal myoblast differentiation is an emerging area of interest in skeletal muscle regeneration research. We have identified differential miRNA expression in differentiating myoblasts exposed to TNF-α or IGF1 treatment. Furthermore, we corroborated prominent muscle differentiation and neuromuscular disease-associated miRNAs. Finally, we identified novel miRNAs which have not previously been associated with skeletal muscle differentiation or response to TNF-α or IGF1. The present study indicates that muscle-regulatory miRNAs are mediators of the inhibitory effect of TNF-α on skeletal muscle differentiation *in vitro*, and suggests that intervention at the miRNA level might be an innovative therapeutic tool to rescue the inhibitory effect of pro-inflammatory TNF-α on myoblast differentiation. Further studies of this beneficial miRNA effect can pave the way for novel intervention strategies in the context of sarcopenia and skeletal muscle disorders.

## Methods

### Cell culture

Primary human skeletal muscle cells (hSkMC), CTRL4107 (Muscle Tissue Culture Collection, Munich, Germany), were propagated from a 34 year-old male’s diagnostic musculus gastrocnemius biopsy (caput laterale) due to non-specific muscular symptoms after informed consent. The muscle biopsy showed no obvious myopathology. HSkMCs were propagated in Skeletal Muscle Cell Growth Medium Low Serum (PromoCell GmbH, Heidelberg, Germany) containing 5% Supplement Mix (PromoCell), 10% foetal calf serum (FCS) (PAA Laboratories GmbH, Pasching, Austria), and 2 mM L-glutamine (PAA Laboratories). HSkMCs cells were plated on laminin-1 coated dishes. After 24 hours, the growth medium was replaced by a differentiation medium (DMEM medium supplemented with 2% horse serum, Life Technologies, Darmstadt, Germany) and 2 mM L-glutamine (PAA Laboratories). Immortalized human skeletal myoblasts LHCN-M2 (LHCN) [26] were propagated in growth medium containing 4 parts DMEM (Life Technologies), 1 part Medium 199, GlutaMAX Supplement (Life Technologies), 20% FCS (Sigma-Aldrich, St. Louis, MO, USA), penicillin (100 I.U./ml)/streptomycin (100 μg/ml) (Life Technologies), and 2.5 μg/ml plasmocin (InvivoGen, San Diego, CA, USA). Prior to the induction of differentiation, cells were seeded on collagen (Sigma-Aldrich) coated plates for 24 hours at 2 ×10^4^ cells/well of a 96-well plate. Differentiation was induced by switching to a differentiation medium composed of DMEM, penicillin (100 I.U./ml)/streptomycin (100 μg/ml), insulin 0.01 mg/ml, and transferrin 0.1 mg/ml (Life Technologies). Cytokine treated-cells received 2 × 10^3^ U/ml human recombinant TNF-α (Roche Diagnostics, Rotkreuz, Switzerland) or carrier for the control. The same amount of TNF-α was added every 24 hours without changing the differentiation medium. LHCN cells were harvested 7 days after the induction of differentiation for immunocytochemistry. For *in vitro* studies on mouse skeletal muscle cells, the murine skeletal myoblast cell line PMI28 [53] was cultured in Ham’s F10 (PAA Laboratories), supplemented with 20% FCS (Sigma-Aldrich), 2 mM L-glutamine (PAA Laboratories), and Penicillin (100 I.U./ml)/Streptomycin (100 μg/ml, PAA Laboratories). PMI28 cells were plated on laminin-1 (Sigma-Aldrich) coated dishes at 1.5 × 10^6^ cells per 10 cm cell culture plate. After 24 hours the growth medium was replaced by a differentiation medium containing DMEM medium with 2% horse serum (Gibco), 2 mM L-glutamine (PAA Laboratories), and Penicillin (100 I.U./ml) / Streptomycin (100 μg/ml) with 2 × 10^3^ U/ml murine recombinant TNF-α (Roche Diagnostics), 5 ng/ml murine recombinant IGF1 (Sigma-Aldrich). For experiments analyzing the influence of MAPK/ERK activity on modulating the effect of TNF-α and IGF1 on miRNA abundance we exposed cells to 20 μM MEK Inhibitor U0126 (Promega Corporation, Madison, WI, USA), or carrier, and combinations of U0126 or carrier and TNF-α or IGF1, respectively. All control and treatment media were replenished twice a day to ensure cytokine and growth factor activity. Murine PMI28 cells were harvested 24 h after the induction of fusion by serum withdrawal for RNA analyses, or after 72 hours for cytochemistry. All cells were propagated at 37°C in 80% relative humidity and 5% CO_2_.

### Cell transfection

Human LHCN cells were transfected with different miRNA inhibitors (miRCURY LNA microRNA Inhibitors) or a scrambled control (miRCURY LNA microRNA Inhibitor Control) from Exiqon (Vedbaek, Denmark) or/and pre-miRNA precursors and precursor scrambled control (mimics) from Ambion (Life Technologies) as follows: The transfection mix for a 96-well plate format contained 10 μl Opti-MEM I Reduced Serum Medium, GlutaMAX Supplement (Life Technologies) per well with final miRNA inhibitor concentrations of 25 nM, 50 nM, or 100 nM, or final miRNA mimic concentrations of 5 nM, 25 nM, or 50 nM as well as Lipofectamine RNAiMAX transfection reagent (Life Technologies). The transfection mix was incubated for 20 minutes at room temperature and added to the well before plating 90 μl of cell suspension. 24 hours after transfection, the growth medium was replenished or the differentiation medium with TNF-α or IGF1 was added, respectively.

### Immuno-fluorescent staining

Differentiation markers of skeletal muscle cells, such as β-dystroglycan or myosin heavy chain were used for immunofluorescence staining and detection of fusion efficiency. Staining against β-dystroglycan was performed as follows: Cells were fixed with 3.7% paraformaldehyde in CSK buffer (100 mM sodium chloride, 300 mM sucrose, 10 mM PIPES [piperazin-1,4-bis (2-ethanesulfonic acid)] pH 6.8, 3 mM magnesium chloride, 1 mM EDTA [ethylene glycol-bis (β-aminoethyl ether)-*N,N,N’,N’*-tetraacetic acid)], washed with 0.15% glycine in phosphate buffered saline (PBS) three times, and blocked with 5% horse serum in PBS. The beta-dystroglycan mouse monoclonal antibody (NCL-ß-DG, Novocastra Laboratories Ltd, Newcastle upon Tyne, United Kingdom) was incubated in a dilution of 1:20. After washing with 0.15% glycine in PBS Cy3-labeled the secondary antibody, anti-mouse IgG (whole molecule) Cy3 conjugate, F (ab’)_2_ fragment of sheep antibody (Sigma-Aldrich), diluted 1:200 was incubated. Cells were washed with 0.15% glycine in PBS with a 20 μg/ml DAPI [4′,6-Diamidino-2-phenylindole dihydrochloride] (Fluka Chemie AG, Neu-Ulm, Germany) and 0.15% glycine in PBS. Afterwards, cells were embedded in a DAKO Fluorescent Mounting Medium (DAKO, Carpinteria, CA, USA). For the automated analysis of 96-well plate format transfection experiments, cells were fixed with 4% paraformaldehyde in PBS, washed with 100 mM glycine in PBS, then washed with PBS and permeabilized with 0.2% Triton X-100 in PBS. After washing with PBS, cells were blocked with IMAGE-iT FX signal enhancer (Life Technologies). For the immunostaining of myosin heavy chain, anti-MHC antibody (Sigma MY-32) was used at a dilution of 1:600. Cells were rinsed with PBS and incubated with the Alexa Fluor 488-labeled secondary anti-mouse antibody (Invitrogen, Life Technologies) diluted to 1:500. After washing with PBS, cells were incubated with 8 μM Hoechst 33258. Washing with PBS preceded the covering of cells with PBS containing 1% paraformaldehyde.

### Microscopy and image analysis

Images of murine skeletal muscle cells PMI28 stained against beta-dystroglycan were recorded by conventional fluorescence microscopy with exposure times of 400 milliseconds for DAPI and 1000 milliseconds for Cy3 staining at 100-fold or 200-fold magnification. Merged-images were analyzed by counting nuclei with the CellProfiler Software [54]. As a measure of terminal differentiation efficiency, the fusion index was calculated as the mean percentage of nuclei in myotubes divided by the total number of nuclei. Myotubes were defined as β-dystroglycan positive structures containing at least three nuclei. Human skeletal muscle cells LHCN used in the transfection experiments were analyzed on an Operetta High Content Screening system (Perkin Elmer, Waltham, MA, USA). Nuclei and myotubes were identified by applying intensity thresholds, area and shape parameters according to Polesskaya et al. [10].

### RNA extraction and quality control

For miRNA profiling, about 2 × 10^6^ cells were harvested in 1.5 ml Trizol (Life Technologies), homogenized, mixed with 0.45 ml chloroform and phase separated by centrifugation. RNA was precipitated by aspiration of the upper aqueous phase, addition of 1.25 ml isopropanol, mixing and centrifugation. The pellet was washed with 75% ethanol, then dried and dissolved in water. Total RNA concentrations were determined photometrically using the NanoDrop 1000 ND-1000 (Peqlab, Erlangen, Germany). Overall RNA quality was assessed on a 1% agarose gel with a 1 KB molecular weight marker separated in parallel. Cells from MEK-inhibitor experiments were lyzed with 700 μl Qiazol (Qiagen, Hilden, Germany), thoroughly mixed with 140 μl chloroform and centrifuged at 12,000 × g at 4°C for 15 minutes. The upper aqueous phase was mixed with 1.5 volumes of ethanol and loaded onto an RNeasy Mini spin column (RNeasy Mini Kit, Qiagen). RNA extraction was performed according to the manufacturer’s instructions.

### Reverse transcription of RNA to cDNA for individual expression analysis

For the analysis of miRNA by individual assays, total RNA was reverse transcribed using the miScript RT Kit (Qiagen) according to the manufacturer’s instructions. For the analysis of mature miRNAs, precursor miRNA and mRNA from the same reverse transcription reaction total RNA was first treated with DNase using the TURBO DNA-free kit (Life Technologies) according to the manufacturer’s instructions prior to reverse transcription.

### MicroRNA profiling by microarray technology

microRNA expression of primary human or murine cell line skeletal muscle cells were performed by using Agilent Human MicroRNA Microarray V2 (8×15K, G4471A-019118 Agilent Technologies, Santa Clara, CA, USA) which contained probes for 723 human microRNAs from the Sanger miRBase v10.1 (www.mirbase.org) and by using Mouse miRNA Microarray Release 15.0 (8×15K, G4471A-029152, Agilent Technologies) which contained probes for 696 miRNAs from Sanger miRBase release 15.0. Quadruplicate or triplicate samples of myoblasts, differentiated myotubes, and TNF-α or IGF1 treated myotubes were included in the study. 100 ng total RNA was used for Cy3-labeling of miRNAs by using the miRNA Complete Labeling and Hybridization Kit (Agilent Technologies) according to the manufacturer’s instructions. Samples were loaded onto the array and hybridized at 55°C for 20 hours. MicroRNA microarrays were washed and scanned with the Agilent Microarray Scanner G2505C in a single pass mode with a scan resolution of 3 μm, 20 bit mode. Signal intensities were extracted and background subtracted using Feature Extraction Software 10.7.3.1 (Agilent Technologies). MicroRNAs which passed the filtering criterion “well above background” in at least two of the replicates within one group were retained. 282 miRNAs met these detection criteria. Data was normalized by loessM normalization [55,56]. Agilent microarray data was MIAME [57] compliant and was registered into the ArrayExpress database (www.ebi.ac.uk/arrayexpress) [58], a publicly available repository consistent with the MIAME guidelines. Data is available with the ArrayExpress accession number E-MTAB-1114 (murine data) or E-MTAB-299 (human data).

### MicroRNA profiling by quantitative qPCR

MicroRNA expression profiling by qPCR was based on TaqMan Array Human MicroRNA Panel v 1.0 (Life Technologies) and TaqMan Rodent MicroRNA Arrays 2.0 (Life Technologies) using TaqMan chemistry according to the manufacturer’s instructions. Three separate reverse transcription reactions per sample were performed. For the murine samples each reverse transcription reaction was preamplified using the Megaplex PreAmp Primers Rodent Pool A and Rodent Pool B (Life Technologies) according to the manufacturer’s instructions. The three separate reverse transcription and preamplification reactions per sample were pooled per original sample and the qPCR reaction mix was prepared according to the manufacturer’s instructions. The array was run on an Applied Biosystems 7900HT Fast Real-Time System with cycling conditions according to the manufacturer’s protocol. Raw expression data was obtained using SDS 2.3 software (Applied Biosystems, Life Technologies). SDS files were loaded into the RQ Manager 1.2 software (Applied Biosystems, Life Technologies). The threshold was manually set to 0.2. Each amplification plot was reviewed manually and the threshold settings were adjusted for individual assays if necessary. MicroRNAs which showed Cq-values smaller than 32 in at least two of the corresponding triplicates of a group were retained for further data processing. Data was normalized by loessM normalization [55,56] using R programming [59].

### Individual quantitative PCR analysis

Individual qPCR reactions were performed for mature and precursor miRNAs using miScript SYBR Green PCR Kit (Qiagen) and the respective miScript Primer Assay (Qiagen) or miScript Precursor Assay (Qiagen) according to the manufacturer’s protocol and were run on a RotorGene Q Real-time PCR cycler (Qiagen).

### Statistics

Significant differences between fusion indices or individual qPCR analyses were determined by using parametric unpaired two-tailed Student’s *t*-test. Relative quantification [60] of miRNA expression levels derived from profiling experiments was tested for significant differences by applying significance analysis of microarrays (SAM) [61] which uses permutation. In addition, false discovery rate (FDR) correction of p-values was performed. Significance analysis and FDR correction were performed by R programming [59].

### Bioinformatics analysis of data

Dynamic principal component analysis (PCA) was performed within GenEx Software (MultiD Analyses AB, Gothenburg, Sweden) comparing myoblasts to the other treatment groups. MiRNAs were filtered by p-values to identify the most relevant genes or miRNAs explaining the observations. The miRSystem [62] bioinformatics tool was applied to identify enriched functions and pathways of miRNA targets using KEGG database information [63].

## Additional files

Additional file 1:
**(Microsoft excel document): miRNA signatures of myoblast differentiation and TNF-α or IGF1 treatment.** Differential miRNA expression is depicted as deltadelta Cq or log2 (signal intensity) for **(A)** murine and **(B)** human miRNA expression profiling by qPCR or microarrays, respectively. Results with p-values <0.05 and regulation >2-fold are depicted in bold. Bold miRNAs indicate cross-platform validation which means that at least one of the treatment effects was validated (significant and >2-fold regulation) on both platforms. One black circle indicates miRNAs detected among the 25% most expressed, two black circles mark miRNAs among the 15% most expressed miRNAs in at least one of the treatment groups on the qPCR array. Some miRNAs are not present on one of the platforms which is indicated by a dash. Some miRNAs could not be detected (n.d.) although present on the platform. Comments or references in parentheses indicate that the information applies to an isoform of the respective miRNA or the corresponding 3p- or 5p-strand instead of the miRNA indicated in the table. Lacking references indicate that, to the best of our knowledge, there was no previous report of differential miRNA expression during skeletal muscle differentiation, TNF-α or IGF1 treatment of skeletal myoblasts or myotubes or in pathological skeletal muscle conditions. Additional file 2:
**(Microsoft word document): Predicted targets of human and murine miRNAs associated with myogenic differentiation, TNF-α or IGF1 response are enriched in selected specific functional annotations.** Murine miRNA targets are enriched in functional annotations during **(A)** myoblast differentiation, **(B)** TNF-α treated, and **(C)** IGF1 treated differentiating myoblasts. Predicted human miRNA targets are enriched in functional annotations during **(D)** myoblast differentiation and **(E)** myoblast differentiation with TNF-α treatment. The complete list can be found in Additional file 4A, B). Additional file 3:
**(Microsoft word document): Human miRNAs associated with myogenic differentiation and TNF-α response are predicted to target genes which are enriched in specific pathways.** Selected KEGG pathways within the top 10 enriched pathways of predicted miRNA targets of differentially regulated human miRNAs. **(A)** KEGG pathways of human myoblast differentiation, **(B)** KEGG pathways of human myoblast differentiation with TNF-α exposure. The complete list can be found in Additional file 4A. Additional file 4:
**(Microsoft excel document): Functional annotation and enrichment of predicted miRNA targets in KEGG, Reactome, Biocarta, Pathway Interaction Database, or gene ontology term molecular function.** Predicted targets of **(A)** human and **(B)** murine miRNAs associated with myogenic differentiation, TNF-α or IGF1 response and their enrichment in specific pathways and functional annotations based on different databases and terms. Additional file 5:
**(Microsoft word document): Differentiation efficiency of skeletal muscle cells is negatively regulated by TNF-α while slightly enhanced by IGF1 treatment.** The fusion efficiency of differentiating murine PMI28 skeletal myoblasts (black bars) on day three of differentiation treated with TNF-α (TNF) or IGF1 (IGF) are represented. Relative fusion indices give the ratio of nuclei in myotubes and total amount of nuclei relative to the control. Fusion indices of human LHCN myoblasts were analysed on day 7 after the induction of differentiation (grey bars). The effect of IGF1 on human LHCN myoblast fusion was not tested. Additional file 6:
**(PNG file): Hierarchical clustering and heatmaps of miRNA expression profiling after 24 h of induction of differentiation and TNF-α or IGF1 treatment.** Hierarchical clustering analysis of **(A)** miRNA microarray profiling data after 24 h of induction of differentiation reveals clear clustering of myoblasts (MB) and most of the myoblast samples treated with TNF-α (TNF). Myoblasts and myotubes (MT) with TNF-α treatment have a large distance to control myotubes and myotubes with IGF1 (IGF), indicating that TNF-α impairs differentiation. **(B)** Hierarchical clustering analysis of miRNA qPCR data reveals separation of the IGF1 treated myotubes from the other samples. Additional file 7:
**(JPEG file): TNF-α and IGF1 may modulate skeletal muscle-related miRNA abundance and biogenesis via MAPK/ERK signalling.** We hypothesized that activation of MAPK/ERK by TNF-α or IGF1 exposure modulates miRNA abundance and biogenesis of skeletal muscle-related miRNAs and myogenic differentiation marker. We postulated that the effects of TNF-α or IGF1 treatment on miRNA expression are distinctly modulated by MAPK/ERK activity. Additional file 8:
**(Microsoft word document): Effect of TNF-α, IGF1, and MAPK/ERK-Inhibitor on mature and precursor miRNA abundance and differentiation marker expression.** qPCR based relative gene expression analysis results of mature and precursor miRNA and selected mRNAs of early murine myoblast differentiation (24 h) treated with TNF-α (TNF), IGF1 (IGF), or MAPK/ERK-inhibitor (MAPK-I) or simultaneous combinations thereof are shown. Mature miRNAs are depicted in black and the sum of primary and precursor (PR) miRNAs is shown in red or green. **(A)** miR-1 (black bar), miR-1-1 precursors (red bar), miR-1-2 precursors (green bar), **(B)** miR-133a (black bar), miR-133a-1 precursors (red bar), miR-133a-2 precursors (green bar), **(C)** miR-206 (black bar), miR-206 precursors (red bar), **(D)** Relative mRNA expression of myogenin (Myog), myocyte enhancer factor 2C (Mef2c), myogenic factor 5 (Myf5), and myosin heavy chain 1 (Myh1). Asterisks indicate significant differential expression with a p-value < 0.05 and more than 1.5-fold change. MAPK-I: MAPK/ERK-inhibitor treatment; MAPK-I (TNF): MAPK/ERK-inhibitor treatment of cells exposed to TNF-α; TNF: TNF-α treatment; TNF (MAPK-I): TNF-α treatment of cells with MAPK/ERK inhibition; MAPK-I (IGF): MAPK/ERK-inhibitor treatment of cells exposed to IGF1; IGF: IGF1 treatment; IGF (MAPK-I): IGF1 treatment of cells with MAPK/ERK inhibition. Gray boxes contain a minus if cells were not exposed to MAPK-I or a cytokine (TNF or IGF). A plus indicates treatment with the respective substance. A bold plus indicates the treatment difference relating to the respective control and thus depicts the measured treatment effect.

## Abbreviation

CTRL4107: Primary human skeletal muscle cells 4107
Dicer: Dicer 1, ribonuclease type III
DMD: Duchenne muscular dystrophy
DMEM: Dulbecco’s modified eagle medium
ErbB: v-erb-b2 erythroblastic leukemia viral oncogene homolog
FCS: Foetal calf serum
Gli1: GLI family zinc finger 1 (*homo sapiens*), GLI-Kruppel family member GLI1 (*mus musculus*)
hSkMC: Primary human skeletal muscle cells
IGF, IGF1: Insulin like growth factor 1
LHCN: A human skeletal muscle precursor cell line/Immortalized human skeletal myoblasts
MAPK/ERK: Mitogen-activated protein kinase 1/extracellular signal-regulated kinase 2
Mef2c: Myocyte-specific enhancing factor 2C
miRNA: Micro ribonuleotide acid
MRF: Myogenic regulatory factorLHCN-M2
Myf5: Myogenic factor 5
Myh1: Myosin heavy chain 1
Myog: Myogenin
NF-κB: Nuclear factor of kappa light polypeptide gene enhancer in B-cells 1
PMI28: A murine skeletal myoblast cell line [53]
PPAR: Peroxisome proliferator-activated receptor
Raf/MEK/ERK axis: v-raf-1 murine leukemia viral oncogene homolog 1 (*homo sapiens*), zinc fingers and homeoboxes 2 (*mus musculus*)/mitogen-activated protein kinase kinase 1/mitogen-activated protein kinase 1
TNF; TNF-α: Tumor necrosis factor alpha
TRBP: TAR RNA binding protein
TWEAK: Tumor necrosis factor (ligand) superfamily, member 12
U0126: MEK Inhibitor U0126
Wnt: Wnt oncogene analog
