# Supplementary material for: TNF-α and IGF1 modify the microRNA signature in skeletal muscle cell differentiation
Source: Cell Commun Signal. 2015 Jan 29;13:4. doi: 10.1186/s12964-015-0083-0 (PMC4325962; doi:10.1186/s12964-015-0083-0)
Supplement: Additional file 2: — (Microsoft word document): Predicted targets of human and murine miRNAs associated with myogenic differentiation, TNF-α or IGF1 response are enriched in selected specific functional annotations. Murine miRNA targets are enriched in functional annotations during (A) myoblast differentiation, (B) TNF-α treated, and (C) IGF1 treated differentiating myoblasts. Predicted human miRNA targets are enriched in functional annotations during (D) myoblast differentiation and (E) myoblast differentiation with TNF-α treatment. The complete list can be found in Additional file 4A, B). [file 12964_2015_83_MOESM2_ESM.docx]

**Additional material 2**
