# Supplementary material for: TNF-α and IGF1 modify the microRNA signature in skeletal muscle cell differentiation
Source: Cell Commun Signal. 2015 Jan 29;13:4. doi: 10.1186/s12964-015-0083-0 (PMC4325962; doi:10.1186/s12964-015-0083-0)
Supplement: Additional file 3: — (Microsoft word document): Human miRNAs associated with myogenic differentiation and TNF-α response are predicted to target genes which are enriched in specific pathways. Selected KEGG pathways within the top 10 enriched pathways of predicted miRNA targets of differentially regulated human miRNAs. (A) KEGG pathways of human myoblast differentiation, (B) KEGG pathways of human myoblast differentiation with TNF-α exposure. The complete list can be found in Additional file 4A. [file 12964_2015_83_MOESM3_ESM.docx]

**Additional material 3**
