# Supplementary material for: TNF-α and IGF1 modify the microRNA signature in skeletal muscle cell differentiation
Source: Cell Commun Signal. 2015 Jan 29;13:4. doi: 10.1186/s12964-015-0083-0 (PMC4325962; doi:10.1186/s12964-015-0083-0)
Supplement: Additional file 5: — (Microsoft word document): Differentiation efficiency of skeletal muscle cells is negatively regulated by TNF-α while slightly enhanced by IGF1 treatment. The fusion efficiency of differentiating murine PMI28 skeletal myoblasts (black bars) on day three of differentiation treated with TNF-α (TNF) or IGF1 (IGF) are represented. Relative fusion indices give the ratio of nuclei in myotubes and total amount of nuclei relative to the control. Fusion indices of human LHCN myoblasts were analysed on day 7 after the induction of differentiation (grey bars). The effect of IGF1 on human LHCN myoblast fusion was not tested. [file 12964_2015_83_MOESM5_ESM.docx]

**Additional material 5**
