# Supplementary figures and images for: TNF-α and IGF1 modify the microRNA signature in skeletal muscle cell differentiation
Source: Cell Commun Signal. 2015 Jan 29;13:4. doi: 10.1186/s12964-015-0083-0 (PMC4325962; doi:10.1186/s12964-015-0083-0)

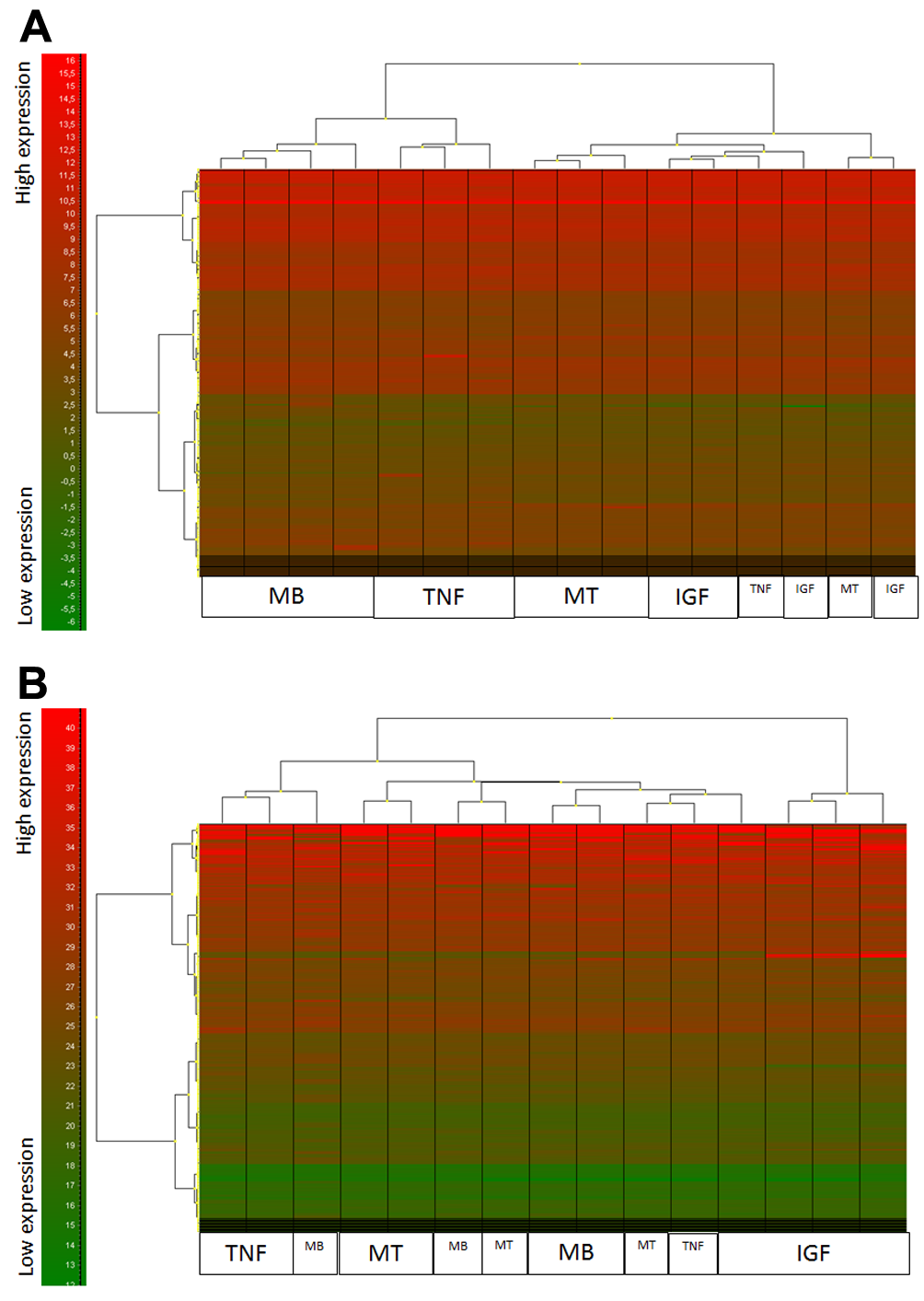

Supplement: Additional file 6: — (PNG file): Hierarchical clustering and heatmaps of miRNA expression profiling after 24 h of induction of differentiation and TNF-α or IGF1 treatment. Hierarchical clustering analysis of (A) miRNA microarray profiling data after 24 h of induction of differentiation reveals clear clustering of myoblasts (MB) and most of the myoblast samples treated with TNF-α (TNF). Myoblasts and myotubes (MT) with TNF-α treatment have a large distance to control myotubes and myotubes with IGF1 (IGF), indicating that TNF-α impairs differentiation. (B) Hierarchical clustering analysis of miRNA qPCR data reveals separation of the IGF1 treated myotubes from the other samples. [file 12964_2015_83_MOESM6_ESM.png]

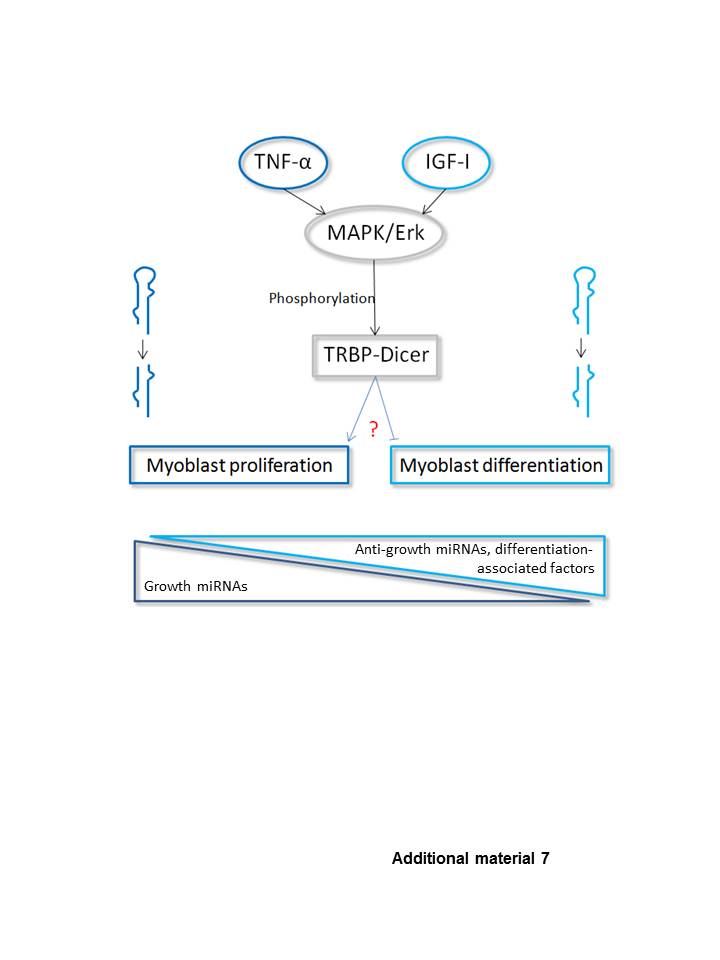

Supplement: Additional file 7: — (JPEG file): TNF-α and IGF1 may modulate skeletal muscle-related miRNA abundance and biogenesis via MAPK/ERK signalling. We hypothesized that activation of MAPK/ERK by TNF-α or IGF1 exposure modulates miRNA abundance and biogenesis of skeletal muscle-related miRNAs and myogenic differentiation marker. We postulated that the effects of TNF-α or IGF1 treatment on miRNA expression are distinctly modulated by MAPK/ERK activity. [file 12964_2015_83_MOESM7_ESM.jpeg]
