# Supplementary material for: TNF-α and IGF1 modify the microRNA signature in skeletal muscle cell differentiation
Source: Cell Commun Signal. 2015 Jan 29;13:4. doi: 10.1186/s12964-015-0083-0 (PMC4325962; doi:10.1186/s12964-015-0083-0)
Supplement: Additional file 8: — (Microsoft word document): Effect of TNF-α, IGF1, and MAPK/ERK-Inhibitor on mature and precursor miRNA abundance and differentiation marker expression. qPCR based relative gene expression analysis results of mature and precursor miRNA and selected mRNAs of early murine myoblast differentiation (24 h) treated with TNF-α (TNF), IGF1 (IGF), or MAPK/ERK-inhibitor (MAPK-I) or simultaneous combinations thereof are shown. Mature miRNAs are depicted in black and the sum of primary and precursor (PR) miRNAs is shown in red or green. (A) miR-1 (black bar), miR-1-1 precursors (red bar), miR-1-2 precursors (green bar), (B) miR-133a (black bar), miR-133a-1 precursors (red bar), miR-133a-2 precursors (green bar), (C) miR-206 (black bar), miR-206 precursors (red bar), (D) Relative mRNA expression of myogenin (Myog), myocyte enhancer factor 2C (Mef2c), myogenic factor 5 (Myf5), and myosin heavy chain 1 (Myh1). Asterisks indicate significant differential expression with a p-value < 0.05 and more than 1.5-fold change. MAPK-I: MAPK/ERK-inhibitor treatment; MAPK-I (TNF): MAPK/ERK-inhibitor treatment of cells exposed to TNF-α; TNF: TNF-α treatment; TNF (MAPK-I): TNF-α treatment of cells with MAPK/ERK inhibition; MAPK-I (IGF): MAPK/ERK-inhibitor treatment of cells exposed to IGF1; IGF: IGF1 treatment; IGF (MAPK-I): IGF1 treatment of cells with MAPK/ERK inhibition. Gray boxes contain a minus if cells were not exposed to MAPK-I or a cytokine (TNF or IGF). A plus indicates treatment with the respective substance. A bold plus indicates the treatment difference relating to the respective control and thus depicts the measured treatment effect. [file 12964_2015_83_MOESM8_ESM.docx]

**Additional material 8**
